# The time course of adaptations in thermoneutral maximal oxygen consumption following heat acclimation

**DOI:** 10.1007/s00421-019-04218-2

**Published:** 2019-09-12

**Authors:** Mark Waldron, O. Jeffries, J. Tallent, S. Patterson, V. Nevola

**Affiliations:** 1 College of Engineering, Swansea University, Swansea, UK; 2 School of Science and Technology, University of New England, Armidale, NSW Australia; 3 School of Biomedical Sciences, Newcastle University, Newcastle Upon Tyne, UK; 4 Sport, Health and Applied Sciences, St Mary’s University, London, UK; 5 Defence Science and Technology Laboratory (Dstl), Fareham, Hampshire UK

**Keywords:** Thermal, Training, Cycling, Endurance, Heat acclimation, Maximal aerobic power

## Abstract

**Purpose:**

This study investigated the effects of a 10-day heat acclimation (HA) programme on the time course of changes in thermoneutral maximal oxygen uptake ($\dot{V}$O_2max_) during and up to 10 days post-HA.

**Methods:**

Twenty-two male cyclists were assigned to a HA or control (Con) training group following baseline ramp tests of thermoneutral $\dot{V}$O_2max_. Ten days of fixed-intensity (50% baseline $\dot{V}$O_2max_) indoor cycling was performed in either ~ 38.0 °C (HA) or ~ 20 °C (Con). $\dot{V}$O_2max_ was re-tested on HA days 5, 10 and post-HA days 1, 2, 3, 4, 5 and 10.

**Results:**

$\dot{V}$O_2max_ initially declined across time in both groups during training (*P* < 0.05), before increasing in the post-HA period in both groups (*P* < 0.05). However, $\dot{V}$O_2max_ was higher than control by post-HA day 4 in the HA group (*P* = 0.046).

**Conclusions:**

The non-linear time course of $\dot{V}$O_2max_ adaptation suggests that post-testing should be performed 96-h post-training to identify the maximal change for most individuals. In preparation for training or testing, athletes can augment their aerobic power in thermoneutral environments by performing 10 days HA, but the full effects will manifest at varying stages of the post-HA period.

## Introduction

Heat acclimation (HA) describes a systematic process, whereby serial exposures to artificially heated environments, often in combination with exercise, can lead to rapid adaptations that enhance the capacity to thermoregulate in the heat (Gibson et al. 2015; Taylor and Cotter 2006). These adaptations improve heat tolerance (Sawka et al. 2011), characterised by increased sudomotor function (Fox et al. 1963), reduced heart rate (HR) (Senay et al. 1976), hypervolemia (Nielsen et al. 1993), increased cardiac output (Lorenzo et al. 2010) and a reduced core body temperature (Tc) for a given heat exposure or exercise intensity (Nielsen et al. 1993). This combination of physiological adaptations can enhance endurance performance in hot (Racinais et al. 2015) and thermoneutral environments (Lorenzo et al. 2010).

The maximal rate of oxygen uptake ($\dot{V}$O_2max_) is an important determinant of endurance performance, explaining ~ 20–60% of the variation in performances of different mode and distance (Coyle et al. 1988; Schabort et al. 2000; Jacobs et al. 2011). $\dot{V}$O_2max_ is chiefly limited by central cardiovascular factors, such as O_2_ transport (Bassett and Howley 2000). Heat acclimation can improve $\dot{V}$O_2max_, and is thought to occur owing to heat-induced cardiovascular changes (Périard et al. 2016). Indeed, there have been historical observations of a relationship between $\dot{V}$O_2max_ and heat tolerance (Shvartz et al. 1978; Pandolf 1979; Havenith and van Middendorp 1990). However, evidence of the efficacy of HA on $\dot{V}$O_2max_ is equivocal, with a number of studies demonstrating 4–13% changes (Nadel et al. 1974; Shvartz et al. 1977; Sawka et al. 1985; Pivarnik et al. 1987; Lorenzo et al. 2010; James et al. 2017) and others reporting no change or a reduction following a range of HA protocols (Houmard et al. 1990; Gore et al. 1997; Chen et al. 2013; Karlsen et al. 2015; Keiser et al. 2015; Neal et al. 2016a, b; Rendell et al. 2017; Sotiridis et al. 2018).

There are various explanations for discrepancies in the aforementioned findings. First, not all studies have evaluated adaptations in $\dot{V}$O_2max_ in thermoneutral conditions, thus limiting the understanding of a transfer between heat-induced adaptation and aerobic capacity in temperate environments, which is a topic of ongoing debate (Nybo and Lundby 2016). This could be related to limited $\dot{V}$O_2max_ trainability (Bouchard et al. 2011) or inter-individual and inter-system differences in adaptation reported after 11 days of isothermal acclimation (Corbett et al. 2018). It is therefore possible that $\dot{V}$O_2max_ increases more substantially among HA-responsive individuals compared to their less responsive counterparts (Minson and Cotter 2016). Daanen et al. (2011) also demonstrated that the acute stress imposed by HA acted, initially, to suppress the adaptation in Tc, before adaptation beyond baseline in the days following acclimation. Thus, a period of recovery (from heat, exercise or both) might be necessary to fully realise adaptation in multi-system physiological measurements, such as $\dot{V}$O_2max_. This reasoning is consistent with the tenets of general adaptation syndrome (Selye 1950), which has been incorporated into exercise training guidelines (ACSM 2009). However, there has been no study to evaluate the detailed time course of change in thermoneutral $\dot{V}$O_2max_ after HA. Therefore, it is possible that previous studies have not: (1) monitored the inter-individual time course of responses of $\dot{V}$O_2max_ to HA across successive days and (2) provided adequate time for adaptation to the combined thermal and exercise training stimuli.

Based on this reasoning, we investigated the effects of a 10-day HA programme on the time course of changes in thermoneutral $\dot{V}$O_2max_, during and up to 10 days post-HA against a control training group. We hypothesised that increases in $\dot{V}$O_2max_ would occur in the HA group in the days following the intervention but presumed variability between individuals in the time course of this response in the post-HA 10-day period.

## Methods

### Participants

Twenty-two healthy, trained amateur male cyclists provided written informed consent to take part in this study. Twelve of the participants (age 23 ± 3 years, stature 1.77 ± 0.61 m, body mass 73.7 ± 4.8 kg, $\dot{V}$O_2max_ 60.8 ± 6.1 ml kg^−1^ min^−1^) were randomly allocated to a HA group, while ten were allocated to a control group (age 25 ± 3 years, stature 1.78 ± 0.46 m, body mass 74.1 ± 5.6 kg, $\dot{V}$O_2max_ 59.8 ± 6.7 ml kg^−1^ min^−1^). All of the participants were habitually training on a weekly basis (13.8 ± 3.3 h·week^−1^) and competing in various amateur cycling events. All of the participants had taken part in outdoor hot weather training in the previous 12 months, while 16 had previously taken part in heat acclimation interventions for varying periods of time over the previous 3 years. However, all participants were deemed to be unacclimatised to heat stress within this study’s HA protocol and they were habitually exposed to only the local environmental conditions or were at least 3 months without exposure. All participants completed a food diary for 2 days prior to each test, which was replicated with similar content and volume for the remainder of the study. The daily average high and low temperatures for the 6 weeks before and during all tests were 12.5 and 3.0 °C, respectively (accuweather.com). The participants were instructed not to use saunas or take hot baths during the study period. Participants were asked to refrain from alcohol and any supplementation during the study period and arrive at the laboratory having eaten a standardised meal and consumed 500 ml of fluid in the previous 2 h. The hydration and pre-meal were chosen by the participants, which we ensured were consistent between visits. Euhydration was verified via urine analysis using an osmometer (< 600 mOsmol kg^−1^ H_2_O, Osmocheck, Vitech Scientific Ltd, UK). Ethical approval was provided by the institutional ethics committee, which was conducted in accordance with the 1964 Helsinki Declaration.

### Design

This study followed an independent groups design (HA vs. Con). After baseline tests of thermoneutral $\dot{V}$O_2max_, the participants were randomly allocated to their groups using a Microsoft Excel random number generator. The HA group visited the laboratory for baseline measurements of $\dot{V}$O_2max_ and completed 10 days of 60-min fixed-intensity (50% $\dot{V}$O_2max_) HA at (38.0 ± 2.4 °C, 30 ± 13% RH) in the following 10-day period. The control group visited the laboratory on the same number of occasions but completed their exercise at the same intensity in controlled conditions (20.0 ± 1.1 °C, 30 ± 4% RH). On days 5, 10 and post-days 1, 2, 3, 4, 5 and 10, thermoneutral (~ 20 °C) $\dot{V}$O_2max_ tests (incremental ramp) were performed to assess the time course of changes during and after HA. Cycling training was continued by all of the participants during the study period, with the volume (mean of 60 min·day^−1^) subtracted from their normal regime. The participants’ training during the study ranged across 3–4 separate sessions, comprising 2–3 long, lower-intensity rides (> 4 h) and 1 higher-intensity interval session. None of the participants cross-trained, with all sessions performed in- or outdoors on a bicycle. In the post-HA period, no training was performed.

#### Incremental ramp tests for $\dot{V}$O_2max_

Participants were familiarised with the cycle ergometer (Monark Exercise AB, Ergomedic 874E, Varberg, Sweden) and saddle and handlebar position were recorded and repeated for all subsequent visits. Participants then completed a 5-min self-selected warm-up prior to completing an incremental ramp test. The test was conducted at self-selected cadences (range 70–95 rev min^−1^), starting at ~ 120 W and increasing by 28–30 W min^−1^ until volitional exhaustion. The same increments were used across all trials, with a mean time to exhaustion of 8.5 ± 1.8 min. Pulmonary gas was measured continuously using a breath-by-breath gas analyser (Jaegar, Oxycon Pro, Viasys Healthcare, Hoechberg, Germany). The gas analyser and flow turbine were calibrated before each test using a known gas mixture (15% O_2_ and 5% CO_2_) and a 3-l syringe, respectively (Hans Rudolph, Kansas City, KS). $\dot{V}$O_2max_ was determined as the mean value recorded over the final 30 s of the test. Criteria for achieving $\dot{V}$O_2max_ was: (1) reaching volitional exhaustion, (2) respiratory exchange ratio > 1.15, (3) final HR within 10 beats min^−1^ of age-predicted maximum and (4) RPE > 19. All criteria were met during the study. The same gas analyser was used throughout the study and calibrated identically. HR was recorded throughout the tests (Polar FT1, Polar Electro Oy, Kempele, Finland). End-power output was measured as the highest external power output reached during the final 1 min of the test. All tests were performed in the morning, prior to HA or thermoneutral training session (Con). In our laboratory, incremental tests of $\dot{V}$O_2max_ have a CV% of 3.0%. The first three $\dot{V}$O_2max_ tests were performed prior to the HA session for that day, in a thermoneutral environment, at the same time of day.

#### Heat acclimation protocol

The power output corresponding to 50% of the participants’ baseline $\dot{V}$O_2max_ was set as the external work intensity for the intervention and was monitored using power output on the cycle ergometer. Cadence was self-selected and adjusted if necessary by the investigators using weights to maintain the target intensity. This intensity was maintained for all subsequent trials but was reduced by 10% if it could not be sustained by the participant. Participants cycled for 60 min per session. This type of HA protocol was selected based on previous studies (Houmard et al. 1990; Senay et al. 1976; Lorenzo et al. 2010; Pandolf et al. 1977). The participants’ nude body mass was recorded pre- and post-session on days 1, 5 and 10 of the HA programme to estimate WBSR by subtracting post-exercise body mass from pre-exercise values (MPMS-230, Marsden Weighing Group, Oxfordshire, UK). A rectal thermometer (Edale Instruments Ltd, Cambridge, UK) was self-inserted 10 cm past the anal sphincter, as an indication of Tc, and recorded every 2 min via a scanning thermometer once inside the heat-controlled chamber (Edale Instruments Ltd, Cambridge, UK). The mean Tc recorded was used for statistical analysis. The participants then entered the heat chamber wearing cycling shorts, socks and training shoes, where they sat upright on the same cycle ergometer used during the ramp test. HR was also recorded, alongside thermal sensation (Ts) at 5-min intervals throughout the exercising protocol. Ts was recorded on an ASHRAE 7-point analogue scale, where − 3 = “very cold”, 0 = “neutral”, and 3 = “very hot” (Zhang et al. 2004). In the control condition, the heat chamber was controlled at 20.0 ± 1.1 °C, 30 ± 4% RH. No fans were used during the exercise trials and no fluid intake was permitted until after the session when post-measures were taken.

#### Plasma volume

On arrival at the laboratory in the morning of HA day 1, HA day 5 and HA day 10 and post-HA day 10, the participants from both the HA and control groups rested in an upright seated position in an air-conditioned room (20 °C and 50% relative humidity) for 15 min. Changes in the concentration of haematocrit (Hct) and haemoglobin (Hb) were subsequently recorded to determine the relative change in plasma volume (Dill and Costill 1974). Capillary blood was drawn from the index finger into two 75 mm hematocrit capillary tubes for duplicate measurements. The whole blood was centrifuged (Hawksley Haematospin 1400 Centrifuge, Hawksley and Sons Ltd., Sussex, UK) for 5 min at 13,000*g*. Post-centrifugation, capillary tubes were analysed for[Hct using a micro-capillary reader (Hawksley and Sons Ltd., Sussex, UK), with the mean of the two measurements reported. All measurements agreed by less than 2%. Capillary blood was taken from the same site for measurement of Hb using a Hemocue Hb 201 + (Hemocue Ltd, Viking Court, Derbyshire, UK). Plasma volume changes (∆%PV) were reported between HA days 1 and 5, HA days 5 and 10 and HA day 10 and post-HA day 10.

### Statistical analysis

Changes in $\dot{V}$O_2max_, end power (2 × 9[time]), ∆%body mass and ∆%PV (2 × 3[time]), Tc, end HR and Ts recorded during the HA programme (2 × 10[time]) were analysed using two-way (group × time) within- and between-analyses of variance. All of the participant characteristics were compared using independent *t* tests, to check for baseline differences. Where relevant, assumptions of sphericity were assessed using Mauchly’s test, with any violations adjusted using the Greenhouse–Geisser correction. When significant *F* values were observed, post hoc tests were used to determine differences. Statistical significance was accepted at *P* < 0.05 for all tests and all analyses were performed on IBM SPSS Statistics (Version 21, IBM Corp., Armonk, NY, USA).

## Results

### Participant characteristics

There were no differences (*P* > 0.05) in the characteristics of the HA and control groups (Table 1). Both groups of participants possessed high $\dot{V}$O_2max_ values relative to their age (Rapp et al. 2018), and followed cycling-specific programmes both habitually (~ 13 to 14 h week^−1^) and during the study (~ 10 to 11 h).

**Table 1:** Participant characteristics for the heat acclimation (HA; *n* = 12) and control groups (*n* = 10)

	HA (mean ± SD)	Control (mean ± SD)
Age (years)	23 ± 3	25 ± 2
Nude body mass (kg)	73.7 ± 4.8	74.1 ± 5.6
$\dot{V}$O_2max_ (ml kg^−1^ min^−1^)	60.8 ± 6.1	59.8 ± 6.7
Annual weekly training (h)	14.0 ± 2.8	13.6 ± 4.1
Previous acclimation (days)^a^	9.7 ± 9.1	4.8 ± 2.7
Training during study (h)	11.3 ± 3.3	10.3 ± 2.7
Training age (years)	4.1 ± 1.8	4.0 ± 1.6

^a^In the previous 3 years

#### $\dot{V}$O_2max_ and end-power output

**Fig. 1:**
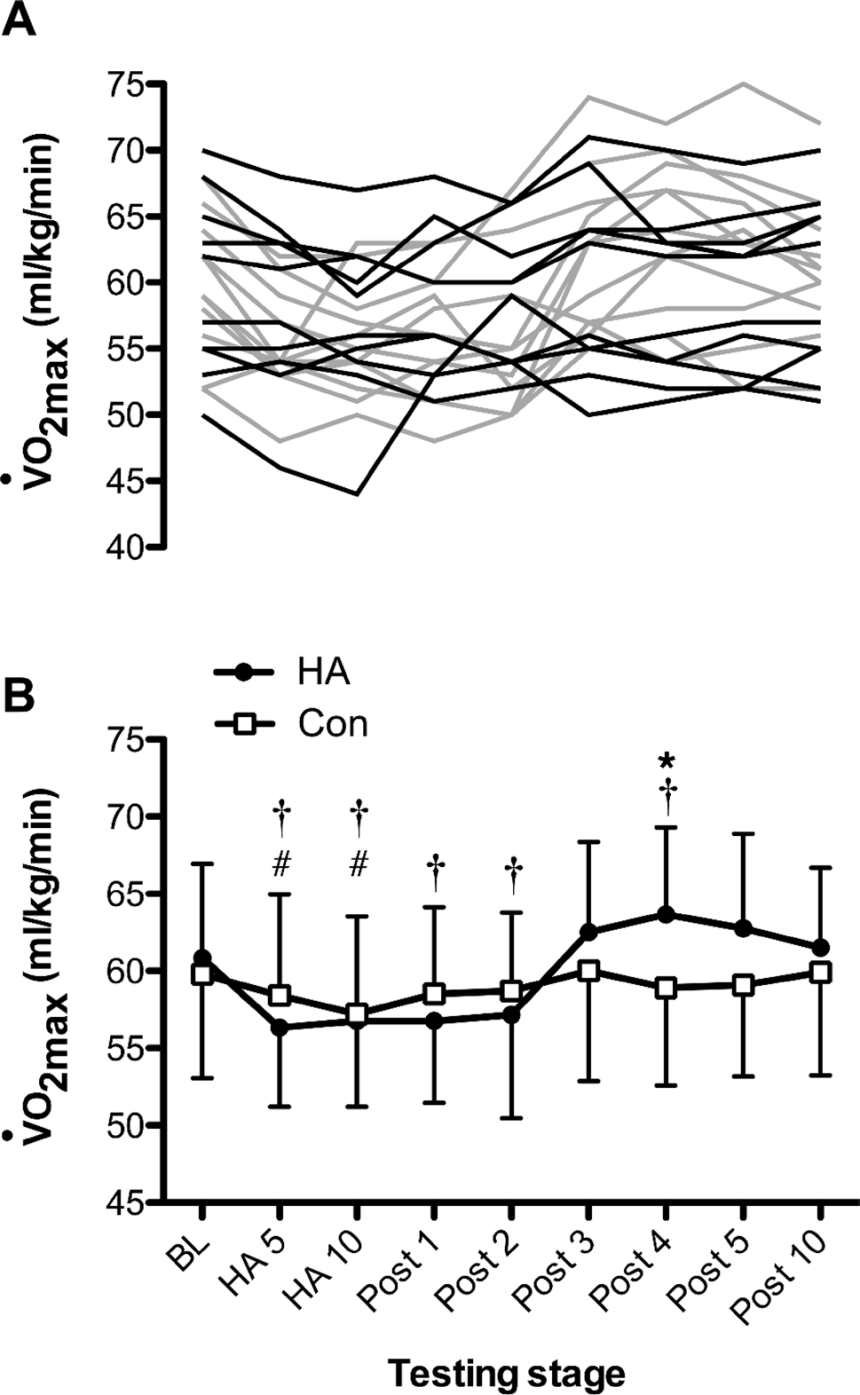
**a** Individual maximal oxygen consumption ($\dot{V}$O_2max_) responses in the HA group (grey) and control (black). **b** Changes in $\dot{V}$O_2max_ across the heat acclimation (HA, *n* = 12) and control (Con, *n* = 10) interventions. *Difference (*P* < 0.05) between groups at that stage; ^†^Sig. different (*P* < 0.05) to baseline (BL) for HA group; ^#^sig. different (*P* < 0.05) to BL for Con group. Time = *P* < 0.001; group × time = *P* < 0.001

**Fig. 2:**
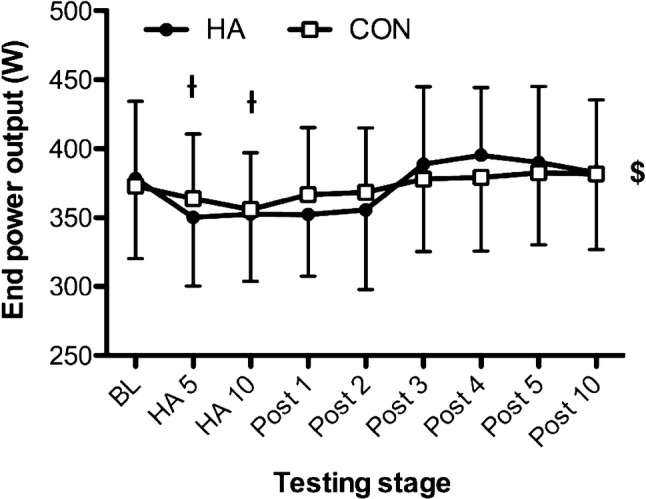
Changes in end-power output across the heat acclimation (HA, *n* = 12) and control (Con, *n* = 10) interventions. ^$^Main effect of time (*P* < 0.05). Time = *P* < 0.001; group × time = *P* = 0.024. ^ƚ^Pairwise time effect from baseline (*P* < 0.05)

Changes in $\dot{V}$O_2max_ across the HA or control programmes are presented in Fig. 1. There was an effect of time on $\dot{V}$O_2max_ [*F*_(8,160)_ = 11.49, *P* < 0.001], and group × time interactions [*F*_(8,160)_ = 5.20, *P* < 0.001], with post hoc tests revealing reductions in $\dot{V}$O_2max_ between baseline and HA days 5 and 10 for the HA group (*P* < 0.001 and *P* = 0.003, respectively) and control (*P* = 0.029 and *P* = 0.032, respectively). However, only the HA group demonstrated reduction in $\dot{V}$O_2max_ compared to baseline on post-HA day 1 (*P* = 0.004), post-HA day 2 (*P* = 0.008), with subsequent increase post-HA day 4 relative to baseline (*P* = 0.038). Across the post-HA period, there were no changes (*P* > 0.05) in $\dot{V}$O_2max_ relative to baseline for the control group. Furthermore, post hoc tests identified differences in $\dot{V}$O_2max_ between the HA group and control group only at post-HA day 4 (*P* = 0.046). As presented in Fig. 2, end-power output followed a similar pattern to $\dot{V}$O_2max_ measurements, with time effects [*F*_(8,160)_ = 13.24, *P* < 0.001], characterised by reductions from BL on HA days 5, 10 and post-HA days 1 (*P* < 0.05). End-power output increased from HA day 10 to post-HA days 3 (*P* = 0.004) and 4 (*P* = 0.004), with no further changes. There were interactions between time and group [*F*_(8,160)_ = 2.33, *P* = 0.024], but no pairwise effects were identified (*P* > 0.05).

#### Core temperature, heart rate and thermal sensation

**Fig. 3:**
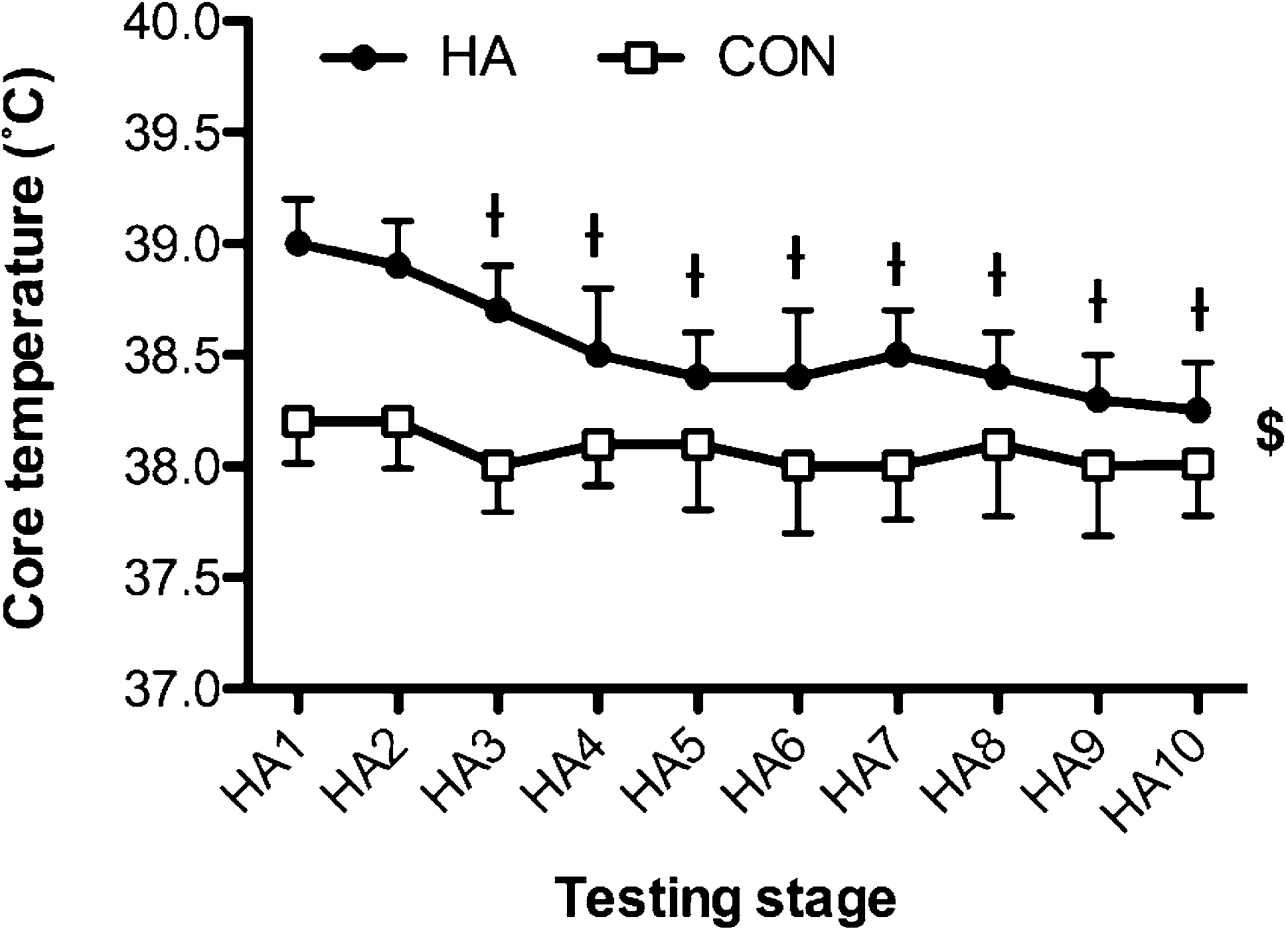
Changes in core temperature across the heat acclimation (HA, *n* = 12) and control (Con, *n* = 10) days. $ = main effect of time (*P* < 0.05). Differences (*P* < 0.05) were found between groups at each stage but not noted for clarity. Time = *P* < 0.001; group × time = *P* < 0.001. ^ƚ^Pairwise time effect from baseline (*P* < 0.05)

**Fig. 4:**
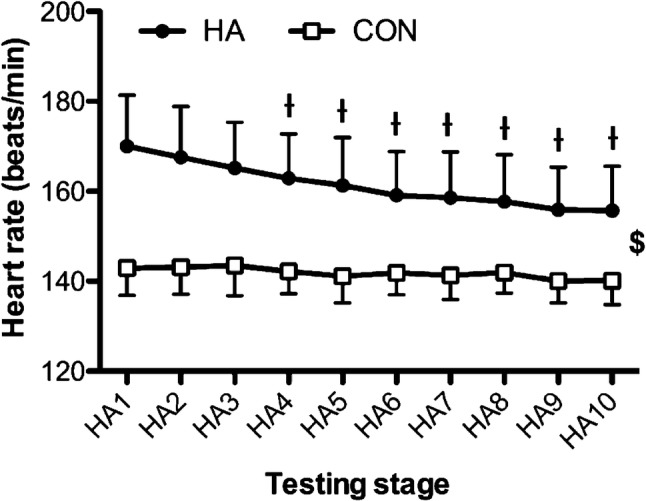
Changes in mean heart rate responses across the heat acclimation (HA, *n* = 12) and control (Con, *n* = 10) days. $ = main effect of time (*P* < 0.05). Differences (*P* < 0.05) were found between groups at each stage but not noted for clarity. Time = *P* < 0.001; group × time = *P* < 0.001. ^ƚ^Pairwise time effect from baseline (*P* < 0.05)

**Fig. 5:**
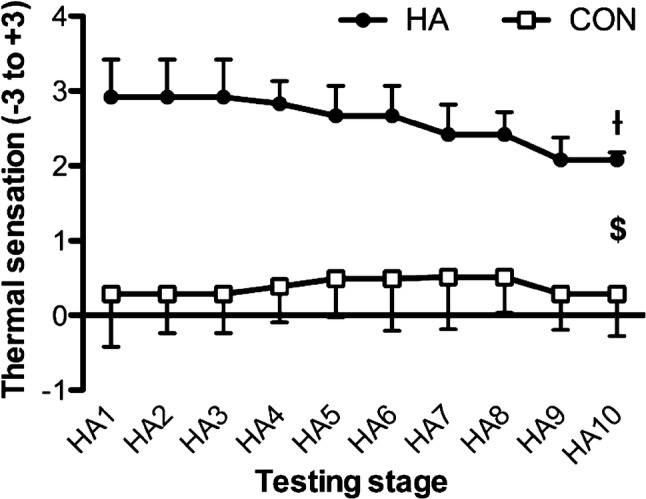
Changes in thermal sensation responses across the heat acclimation (HA, *n* = 12) and control (Con, *n* = 10) days. ^$^Main effect of time (*P* < 0.05). Differences (*P* < 0.05) were found between groups at each stage but not noted for clarity. Time = *P* < 0.001; group × time = *P* = 0.045. ^ƚ^Pairwise time effect from baseline (*P* < 0.05)

There were time effects for mean Tc [*F*_(9,180)_ = 26.93, *P* < 0.001], with step-wise reductions from day 1 across the three subsequent HA days (*P* < 0.05). The main contributions to the time effects were from the HA group, demonstrated via interactions [*F*_(9,180)_ = 11.71, *P* < 0.001] and pairwise differences across all days (*P* < 0.05) (Fig. 3). Mean heart rate [*F*_(9,180)_ = 38.90, *P* < 0.001] and thermal sensation [*F*_(9,180)_ = 5.56, *P* < 0.001] followed a similar pattern across time. Time and group interacted [*F*_(9,180)_ = 17.07, *P* < 0.001] for heart rate responses, with pairwise differences across all HA days (Fig. 4). Similarly, group × time interactions [*F*_(9,180)_ = 2.69, *P* = 0.045] were found for Ts and pairwise differences across all HA days (*P* < 0.05) (Fig. 5).

#### Plasma volume and body mass changes

The ∆%PV between HA days 1 and 5 was larger (*P* < 0.001) in the HA group compared to control (8.8 ± 5.0% vs*.* 0.7 ± 0.8%, respectively). There were no further between-group differences (*P* > 0.05) in ∆%PV, with the HA group maintaining their initial PV expansion between HA days 5 and 10 (HA 1.1 ± 2.6% vs*.* Con − 1.2 ± 1.3%) and HA day 10 and post-HA day 10 (HA 0.6 ± 2.1% vs*.* Con − 1.11 ± 1.5%). There was an effect of group for ∆%body mass changes [*F*_(1,20)_ = 30.05, *P* < 0.001], denoting greater WBSR in the HA group, but no interactions with time [*F*_(2,40)_ = 0.25, *P* = 0.068].

## Discussion

We are the first to evaluate the time course of changes in thermoneutral $\dot{V}$O_2max_ adaptation during and after a HA programme. Changes in $\dot{V}$O_2max_ were elicited in a non-linear manner during the 20-day (combined HA and post-HA) period, with acute reductions apparent at HA days 5 and 10 among both groups. Delayed increase in thermoneutral $\dot{V}$O_2max_ occurred in the post-HA (recovery) period. Specifically, by post-HA day 4, there were pairwise differences between the HA and control groups, where mean changes in $\dot{V}$O_2max_ peaked (4.9% increase from baseline) for the HA group. The control group’s $\dot{V}$O_2max_ fluctuated between − 4.5 and 0.3% approximating the 3.0% error of the test. There were notable inter-individual differences in the time course of adaptation among the HA group, yet only 1 of the 12 participants did not respond to the HA programme, demonstrating their highest value prior to the post-HA period. These adaptations were supported by larger plasma volume expansion in the HA group, which was maintained across the study period.

There are a number of studies demonstrating HA-induced increase in thermoneutral $\dot{V}$O_2max_, ranging between 4 and 13% (Nadel et al. 1974; Shvartz et al. 1977; Sawka et al. 1985; Pivarnik et al. 1987; Lorenzo et al. 2010; James et al. 2017). Similarly, we report a 4.9% increase on post-HA day 4 compared to baseline, which is substantial, given the limited potential for adaptation in $\dot{V}$O_2max_ (Bouchard et al. 2011) and the trained status of the participants. Furthermore, this value was increased to a mean of 7.1% if the largest individual changes in $\dot{V}$O_2max_ observed across the study are accounted for. These data suggest that the changes reported previously are likely to have underestimated the full adaptation potential, owing to inter-individual variability in this measure. Our findings highlight that the timing of the post-acclimation testing is crucial to the outcome, despite most studies not precisely reporting this. Based on the current data, we recommend that post-acclimation $\dot{V}$O_2max_ testing is not performed within the post-HA 48 h and that a 96-h post-test is likely to produce the highest value for most individuals. Of note, the one participant’s $\dot{V}$O_2max_ that did not peak in the post-HA period was highest at baseline and remained lower for the entire 20-day period. This denotes an important individual feature of training adaptations and questions rigid training models. As reported elsewhere, it is also possible that some individuals experience a delayed response (absence of decay) to heat acclimation (Daanen et al. 2011) of up to 26 days (Weller et al. 2007), which would not have been captured in our 10-day post-HA window and is one explanation for the participant’s response in the current study. This descriptive analysis provides insight into the dynamics of heat-induced adaptation in $\dot{V}$O_2max_ between individuals and could lead to imprecise reporting in studies of this type, depending on the selected post-testing period.

As noted previously (Armstrong and Maresh 1991; Nielsen et al. 1993), there were some characteristic signs of early adaptation demonstrated across the sub-maximal HA sessions, with Tc, HR and Ts declining across the first 3 days in the HA group. These changes indicate an improved tolerance to the exercise and thermal stimuli. However, the time course of $\dot{V}$O_2max_ adaptation to HA was not as immediate and, therefore, did not coincide with early adaptations in Tc, HR and Ts. This is important to recognise because HA-induced changes in $\dot{V}$O_2max_ are typically ascribed to central cardiovascular adaptations that are responsible for O_2_ transport. For example, $\dot{V}$O_2max_ is chiefly determined by the delivery of oxygenated blood to the working musculature and relies on factors, such as stroke volume and muscle capillarisation, to support this (Bassett and Howley 2000). The lower HR responses to the HA programme reported here and elsewhere (Nielsen et al. 1993; Lorenzo et al. 2010), and increased plasma volume, are consistent with an improved cardiac efficiency, which is commonly observed following HA (Périard et al. 2016). These changes also infer an increase in stroke volume and cardiac output (Senay et al. 1976; Nielsen et al. 1997). Thus, the temporal differences in $\dot{V}$O_2max_ adaptation, highlighted by acute decline in $\dot{V}$O_2max_, must be attributed to other factors in the O_2_ transport pathway, perhaps indicating an acute impairment and perfusion capacity of muscle microvasculature function during the HA programme. There have been reports of increased vascular conductance after 10–14 days of HA in the peripheral cutaneous microvasculature (Lorenzo and Minson 2010) and others have reported changes in O_2_ pulse at high exercise intensities, which were ascribed to increase in arterial–venous O_2_ differences (Chen et al. 2013). Together, these findings indicate that HA induces an increase in peripheral blood flow that could lead to enhanced perfusion of blood in the working muscles. However, there has been no study of the time course of this adaptation to monitor early-phase changes in the microvasculature of the skeletal muscle. Further work is required to elucidate this.

$\dot{V}$O_2max_ relies on multiple physiological factors; therefore, a less predictable time course of adaptation might have been anticipated. General adaptation syndrome describes a process of systematic overload to impose controlled ‘stress’, leading to changes in that system, such that subsequent exposures to the same stress are more tolerable (Selye 1950). Indeed, the discordant temporal adaptations between different physiological processes observed here are supported by Selye’s (1938) concept of ‘adaptation energy’, whereby simultaneous adaptations to given stimuli are constrained by finite energy resources, which was originally demonstrated by exposing rats to cold temperatures and exercise. Similar adaptation kinetics have been demonstrated in the exercise training literature, where 1–3 weeks of overreaching in cyclists acutely reduced $\dot{V}$O_2max_ prior to rebound improvements in the recovery period (Jeukendrup et al. 1992; Aubry et al. 2014). These changes have not been fully explained but attributed to a general training fatigue or psychological factors that ensue during heavy training periods. This theory has also been previously applied to the process of HA, where Daanen et al. (2011) reported lower resting and exercising Tc—a principal feature of adaptation to heat exposure—to manifest most prominently during the post-HA period. This had been reported in other investigations (Pandolf et al. 1977) and was explained by the severity of the imposed thermal stimulus. It is plausible that the same reasoning applies to the current data, since the participants trained for the duration of the study, in addition to the thermal load and serial $\dot{V}$O_2max_ testing. However, the absence of significant $\dot{V}$O_2max_ changes over time in the control group or differences in training characteristics compared to the HA group suggests that the thermal stimulus was the responsible factor. Alternatively, it is possible that kinetics of $\dot{V}$O_2max_ adaptation are different to other measures but has not yet been considered with the necessary scrutiny.

The suggestion that $\dot{V}$O_2max_ adaptation occurs in the post-HA period is at odds with the characteristic rapid decay reported in other physiological measures, such as Tc and HR (Garrett et al. 2009, 2011; Daanen et al. 2018). Indeed, it is the concern over rapid post-HA decay that has most likely prompted testing in the following 72-h period (i.e., Sotiridis et al. 2018). Of note, some studies have reported descriptive increase in $\dot{V}$O_2max_ of up to 3.5% and significant increase in end test power of 8.5% (Sotiridis et al. 2018), yet re-tested across 3 consecutive days, immediately following HA. Based on the current data, $\dot{V}$O_2max_ does not appear to enter a period of sustained ‘decay’ for the first 4 days, and perhaps up to 10 days, after HA. Future studies should focus on manipulating the intensity of the stimulus and extend the period of post-HA monitoring to establish the full time course of decay in $\dot{V}$O_2max_. In this regard, the chosen HA protocol followed a so-called ‘fixed-intensity’ model, which is not thought to provide an optimal stimulus for adaptation to the heat, as Tc is uncontrolled by the investigator (Daanen et al. 2018). Indeed, others have suggested that acclimation > 38.5 °C Tc is necessary for adaptation (Fox et al. 1963). However, recent studies have questioned this recommendation, reporting no relationship between thermal load (time spent > 38.5 °C) and changes in Tc or HR (Corbett et al. 2018). Others have used fixed-intensity HA programmes to induce changes in $\dot{V}$O_2max_ (Lorenzo et al. 2010) and more recent studies have demonstrated similar heat adaptations between isothermal or fixed-intensity approaches (Gibson et al. 2015). There are some potential advantages to adopting a fixed-intensity protocol. For example, the absolute power output and relative intensity sustained in a fixed-intensity protocol is higher than reported in isothermal models (Gibson et al. 2015), which might be of greater importance when a gross outcome ($\dot{V}$O_2max_) is targeted, requiring a mixture of thermal and exercise stimuli. The Tc and HR responses were also significantly higher than the control group across all sessions, with mean Tc values beginning at a mean of 39 °C and finishing marginally above 38.2 °C on day 10. Furthermore, this model can be practically simpler to run with groups of athletes, which is often necessary for applied practitioners. Therefore, the approach we have adopted appears to have provided sufficient stimulus for adaptation in $\dot{V}$O_2max_ and the delayed increase could, theoretically, suit the needs of individuals who cannot acclimate for the entire pre-competition training camp, owing to travel arrangements or the details of their pre-event taper.

There are some limitations to the current study, such as the types of athletes used, who were all cyclists and well trained. While we do not anticipate that delayed $\dot{V}$O_2max_ adaptation will be specific to the mode of exercise, it is possible that less trained participants would demonstrate a different time course of adaptation to training stimuli and that the lower aerobic capacity might prevent them from fully engaging in the intensive acclimation process. Furthermore, all of the cyclists underwent an acclimation intervention, rather than acclimatisation, where there is typically less control over the ambient conditions (i.e., heat, humidity and wind speed). Thus, we cannot be certain that the same effects would be observed in a natural environment.

## Conclusion

The time course of $\dot{V}$O_2max_ adaptation is non-uniform, mimicking a typical supercompensation response. These findings have implications for researchers and athletes wishing to evaluate adaptations in $\dot{V}$O_2max_ after performing HA programmes. We advise that post-testing is not performed in the following 48 h and that 96 h provides the peak adaptation for most individuals. Whilst the current findings are of fundamental scientific interest, there are also many practical advantages of using heat acclimation to enhance thermoneutral aerobic capacity. This includes the ability to induce a variety of physiological adaptations (reduced Tc and HR, increased sweat response), alongside changes in $\dot{V}$O_2max_, in response to a training programme.

## Abbreviations

CO_2_: Carbon dioxide
Con: Control
Tc: Core body temperature
HA: Heat acclimation
HR: Heart rate
Hct: Hematocrit
Hb: Haemoglobin
$\dot{V}$O_2max_: Maximal oxygen uptake
PV: Plasma volume
RH: Relative humidity
RPE: Rating of perceived exertion
Ts: Thermal sensation
